# Pathological mechanism of chondrocytes and the surrounding environment during osteoarthritis of temporomandibular joint

**DOI:** 10.1111/jcmm.16514

**Published:** 2021-05-05

**Authors:** Baochao Li, Guangzhao Guan, Li Mei, Kai Jiao, Huang Li

**Affiliations:** ^1^ Department of Orthodontics Nanjing Stomatological Hospital Medical School of Nanjing University Nanjing China; ^2^ Department of Oral Sciences Sir John Walsh Research Institute Faculty of Dentistry University of Otago Dunedin New Zealand; ^3^ State Key Laboratory of Military Stomatology & National Clinical Research Center for Oral Diseases & Shaanxi Key Laboratory of Stomatology School of Stomatology The Fourth Military Medical University Xi'an, Shaanxi China

**Keywords:** angiogenesis, bone remodelling, cartilage degeneration, chondrocyte hypertrophy, osteoarthritis, temporomandibular joint

## Abstract

Temporomandibular joint (TMJ) osteoarthritis is a common chronic degenerative disease of the TMJ. In order to explore its aetiology and pathological mechanism, many animal models and cell models have been constructed to simulate the pathological process of TMJ osteoarthritis. The main pathological features of TMJ osteoarthritis include chondrocyte death, extracellular matrix (ECM) degradation and subchondral bone remodelling. Chondrocyte apoptosis accelerates the destruction of cartilage. However, autophagy has a protective effect on condylar chondrocytes. Degradation of ECM not only changes the properties of cartilage but also affects the phenotype of chondrocytes. The loss of subchondral bone in the early stages of TMJ osteoarthritis plays an aetiological role in the onset of osteoarthritis. In recent years, increasing evidence has suggested that chondrocyte hypertrophy and endochondral angiogenesis promote TMJ osteoarthritis. Hypertrophic chondrocytes secrete many factors that promote cartilage degeneration. These chondrocytes can further differentiate into osteoblasts and osteocytes and accelerate cartilage ossification. Intrachondral angiogenesis and neoneurogenesis are considered to be important triggers of arthralgia in TMJ osteoarthritis. Many molecular signalling pathways in endochondral osteogenesis are responsible for TMJ osteoarthritis. These latest discoveries in TMJ osteoarthritis have further enhanced the understanding of this disease and contributed to the development of molecular therapies. This paper summarizes recent cognition on the pathogenesis of TMJ osteoarthritis, focusing on the role of chondrocyte hypertrophy degeneration and cartilage angiogenesis.

## 1 INTRODUCTION

Temporomandibular joint (TMJ) osteoarthritis is a progressive degenerative cartilage disease that affects cartilage and subchondral bone.^1^, ^2^ Chondrocyte death, extracellular matrix (ECM) degradation and subchondral bone remodelling are considered to be the main characteristics of TMJ osteoarthritis.^3^, ^4^ The characteristic progressive breakdown of cartilage results from the abnormal regulation of chondrocytes and the imbalance between the degradation and formation of tissue.

Chondrocytes maintain balance between synthesis and degradation of the ECM.^5^ However, this homeostasis can be disrupted by the uncoupled catabolic and synthetic activities.^6^ Moreover, the number of apoptotic chondrocytes increases significantly in the osteoarthritis, which is closely related to the endoplasmic reticulum and death receptor pathways.^7^, ^8^ The quality of condylar bone is closely related to the development of TMJ osteoarthritis. When TMJ osteoarthritis occurs, the density of subchondral bone is reduced.^4^, ^9^

**FIGURE 1:**
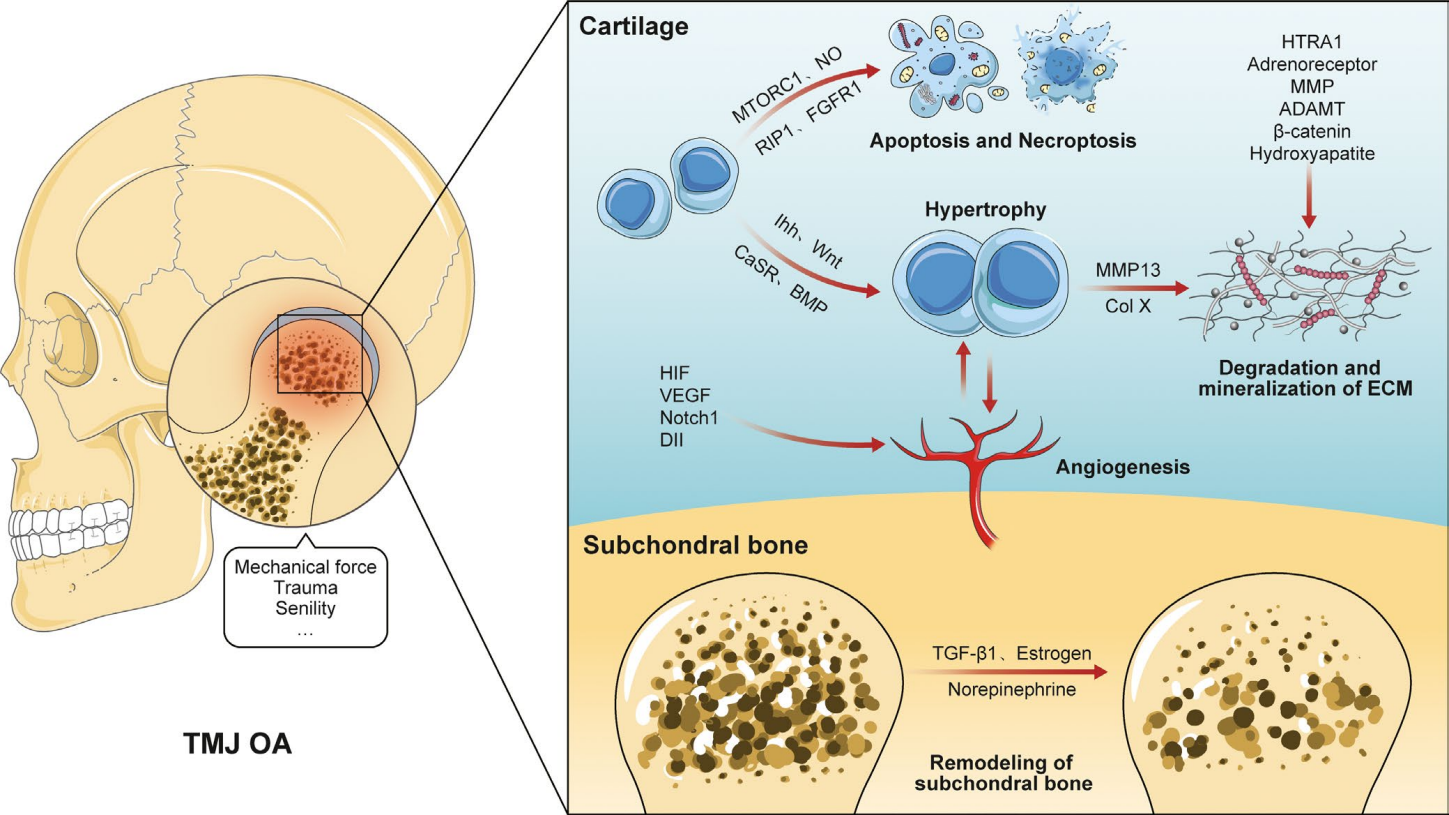
The main pathological changes in TMJ osteoarthritis

The mechanism and role of chondrocyte hypertrophy and cartilage angiogenesis in TMJ osteoarthritis have attracted the attention of researchers. Hypertrophic chondrocytes promote ECM degradation and cartilage calcification. This denatured cartilage shows less mechanical adaption to adverse stimuli such as trauma.^10^ Apart from hypertrophic chondrocytes, angiogenesis has been reported to participate in TMJ osteoarthritis.^11^ Neo‐angiogenesis has been shown to exacerbate chronic pain and promote cartilage ossification.^12^, ^13^ However, the exact mechanism is unclear. The possible pathogenesis of TMJ osteoarthritis is summarized in Figure 1.

In this review, we summarize the latest studies on the pathogenesis of TMJ osteoarthritis and analyse the pathological changes with an emphasis on chondrocyte hypertrophy and angiogenesis.

## 2 IN VIVO ANIMAL MODELS AND IN VITRO CELL MODELS OF TMJ OSTEOARTHRITIS

Animal studies of the development of TMJ osteoarthritis used techniques including modifying the occlusal state, gene editing, injecting inflammatory mediators and surgery (Table 1).

**TABLE 1:** Animal models and cell models used to mimic TMJ osteoarthritis in recent years

Intervention	Animals/Cells	Methods	Year	Authors
In vivo				
Mechanical modification	Rats	Unilateral anterior crossbite	2019	Yang, H., et al
			2018	Ye, T., et al
		Bite‐raising	2019	Long, HQ, et al
		Steady mouth‐opening	2017	Ge, X., R., et al
		External compressive mechanical force	2017	Zhang, C., et al
			2016	Zhu, M., et al
	Rabbits	Unilateral dental splints	2015	Henderson., et al
Medicine	Rats	Monosodium iodoacetic acid	2019	Zhang, S., et al
		Freund's complete adjuvant	2016	Xu, L., et al
	Rabbits	Type II collagenase	2020	Yi, X., et al
Surgery	Mice	Unilateral partial discectomy	2019	Chen, PH et al
	Goats	Total disc removal	2017	Lan, L., et al
		Cartilage removal of condyle	2017	Wang, F., et al
Gene	Mice	Collagen type XI haploinsufficient mice	2019	Chen, PH, et al
		Camurati–Engelmann disease mice	2018	Zheng, L., et al
		β‐catenin conditional activation mice	2018	Hui, T., et al
		Expressing human SHOX	2016	Liang, W., et al
		Conditional deletion of FGFR3	2016	Zhou, S., et al
		SMAD3 deficiency mice	2015	Mori, H., et al
	Guinea pig	Dunkin‐Hartley strain guinea pig	2016	Wu, M., et al
Senescence	Mice		2018	Wang, Z., et al
In vitro				
Mechanical force	ATDC5	Flow fluid shear stress	2019	Yang, H., et al
	Pig condylar chondrocytes		2017	Zhang, M., et al
Cytokines	Rat condylar chondrocytes	IL‐1β	2019	Zhang, S., et al
			2016	Chen, H., et al
		TNF‐α+cycloheximide	2017	Zhang, C., et al

Mechanical modification of the occlusal state is the most commonly used method to induce TMJ osteoarthritis‐like changes. The TMJ receives direct occlusal loading, and disruption of occlusal harmony may traumatize the TMJ during functional loading.^14^ Intraoral occlusal devices such as unilateral anterior crossbite (UAC) and bite‐raising plates have been used to induce TMJ osteoarthritis‐like lesions.^7^, ^15^, ^16^ In addition, an extraoral compression mechanical force device that directly exerts compressive pressure on the TMJ has also been used.^8^, ^17^

Similar to other osteoarthritis animal models, gene editing such as overexpression of short stature homeobox 2 (SHOX), transforming growth factor‐β1 (TGF‐β1), and β‐catenin,^18^, ^19^, ^20^, ^21^ and inhibiting or knocking out genes such as Discoidin domain receptor 1 (DDR1), small mother against decapentaplegic 3 (SMAD3), and fibroblast growth factor receptor (FGFR3), could lead to the formation of TMJ osteoarthritis.^1^, ^22^, ^23^ Intra‐articular injection of chemical mediators such as monosodium iodoacetic acid (MIA) and complete Freund's adjuvant (CFA) could mimic the inflammatory response of the TMJ over a short period of time.^24^, ^25^, ^26^ Surgical methods have been used to destroy part or all of the disc or cartilage of the condyle.^27^, ^28^, ^29^ In addition, since the occurrence of osteoarthritis is closely related to age,^30^ aged mice were used in some studies to mimic spontaneous TMJ osteoarthritis.^31^

In vitro (Table 1), primary chondrocytes could be directly extracted from TMJ osteoarthritis patients or model animal and used as cell models. Chondrocyte inflammation can also be induced by inflammatory chemical mediators such as IL‐1β,^32^, ^33^ and mechanical devices such as Flexcell.^34^, ^35^

## 3 THE CANONICAL PATHOLOGICAL CHANGES DURING THE TMJ OSTEOARTHRITIS

### 3.1 The death of chondrocytes

**FIGURE 2:**
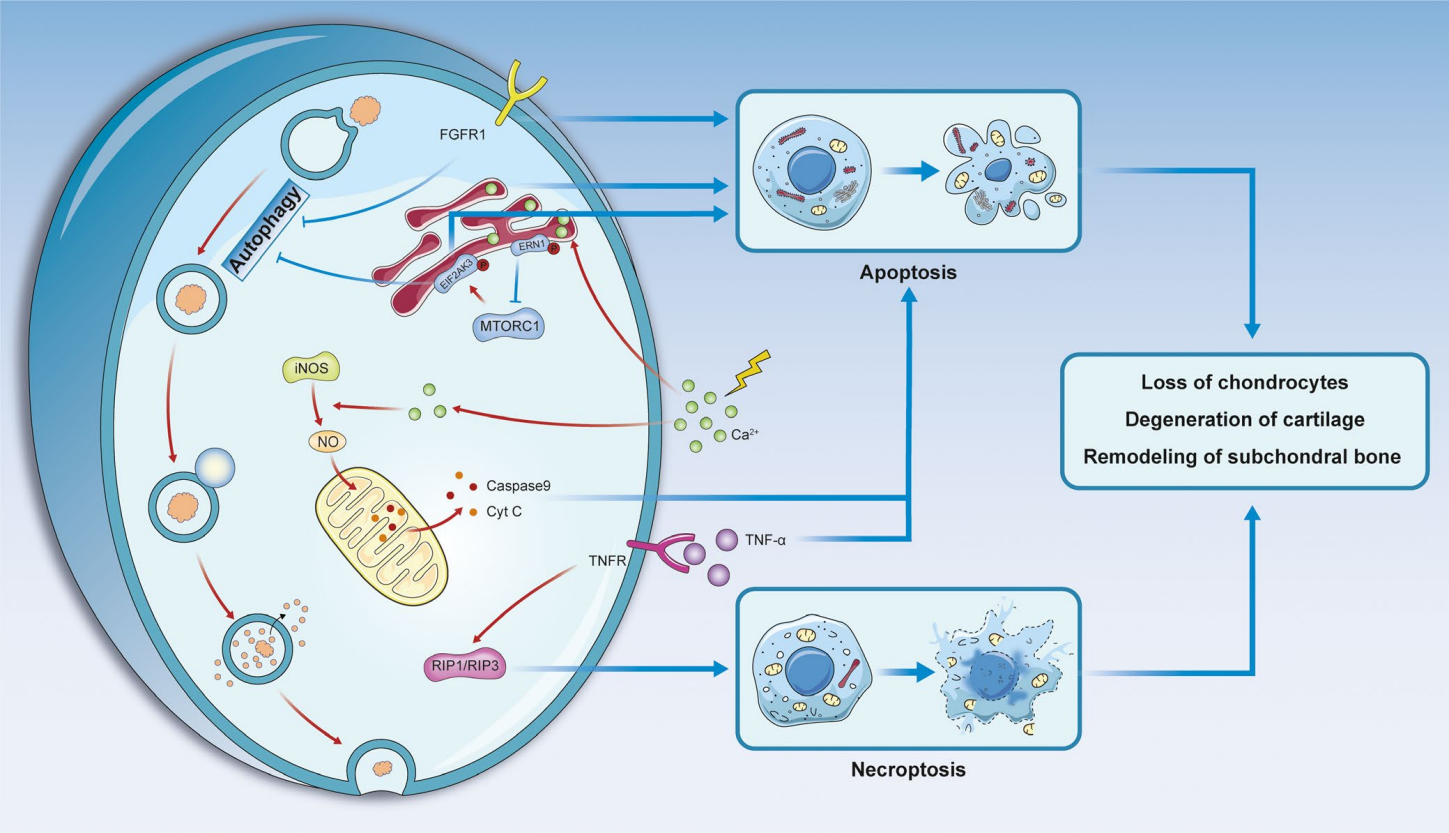
Pathways associated with chondrocyte death in TMJ osteoarthritis. Apoptosis and necroptosis are promoted in TMJ osteoarthritis, but the general trend of autophagy activity is inhibited

Several studies have demonstrated that increased turnover of chondrocytes initiates the degeneration of condylar cartilage.^36^ Apoptosis, autophagy, and necroptosis, play crucial roles in chondrocyte death. Apoptosis and necroptosis accelerate the destruction of articular cartilage.^37^, ^38^ Chondrocyte apoptosis provides space for neovascularization, and the apoptotic bodies produced by this process are thought to be the source of cartilage mineralization.^10^, ^39^ Autophagy is considered a self‐protective mechanism, by which chondrocytes recycle or reuse large biomolecules.^40^ In general, abnormal death of chondrocytes not only reduces the number of chondrocytes, but also initiates the degeneration of cartilage and the destruction of subchondral bone.^41^ The pathways associated with chondrocyte death in TMJ osteoarthritis are illustrated in Figure 2.

#### 3.1.1 Apoptosis and necroptosis in TMJ osteoarthritis

Calcium plays an essential role in chondrocyte apoptosis and calcium concentration in chondrocytes can be increased under mechanical overload.^42^ Endoplasmic reticulum stress (ERS) caused by calcium influx can cause chondrocyte apoptosis, which is known as ERS‐mediated apoptosis.^17^, ^35^, ^42^ A high intracellular calcium concentration can activate inducible nitric oxide synthase (iNOS). Nitric oxide (NO) induced by iNOS inhibits mitochondrial respiration and leads to chondrocyte apoptosis through the release of cytochrome C (Cyt C) and caspase‐9.^43^ Furthermore, tumour necrosis factor (TNF) and FGFR1 are crucial cytokines that can facilitate chondrocyte apoptosis by acting on the death receptor pathway.^44^, ^45^

Necroptosis is mediated by oxidative stress and plays an important role in osteoarthritis.^8^ TNF and receptor‐interacting protein 1 and 3 (RIP1/RIP3)‐mediated necroptosis exacerbates the cartilage destruction. Studies have shown that the necroptosis pathway is enhanced when apoptosis is inhibited.^8^, ^46^

#### 3.1.2 Autophagy in TMJ osteoarthritis

Autophagy is an important mechanism of chondrocyte survival in osteoarthritis.^40^ The main process of autophagy involves the formation of autophagosomes, which sequester discarded organelles or macromolecules. Autophagosomes then fuse with lysosomes to form autolysosomes, which eventually degrade the contained materials and release small molecules that can be reused.^47^ The markers of autophagy, beclin 1 and light chain 3 beta (LC3B) increase in the early stage of TMJ osteoarthritis but decrease significantly in the late stage.^7^ The initial enhancement in autophagy protects chondrocytes from various environmental changes. With the destruction of cartilage, inhibition of autophagy is associated with cell death.^48^ In models of abnormal dental occlusion and age‐associated spontaneous osteoarthritis, the loss of FGFR1 inhibits the development of osteoarthritis by promoting autophagic activity.^49^ In addition, the endoplasmic reticulum‐associated proteins endoplasmic reticulum to nucleus signalling 1 (ERN1), mechanistic target of rapamycin kinase complex 1 (MTORC1) and eukaryotic translation initiation factor 2 alpha kinase3 (EIF2AK3) not only induce apoptosis but also act as a regulatory valve to inhibit autophagy. This pathway is known as the ERN1‐MTORC1‐EIF2AK3 signalling axis.^7^ Therefore, regulating autophagy may be an effective mechanism for the treatment of TMJ osteoarthritis.

### 3.2 Degenerative changes in the ECM

The ECM of cartilage is mainly composed of collagen fibres and large proteoglycans. It not only acts as a protective scaffold against elastic and shear forces for cartilage, but also regulates the behaviour of chondrocytes through matrix‐cell interactions.^5^, ^50^

In TMJ osteoarthritis, ECM degradation starts with expression of matrix metalloproteinases (MMPs) and a disintegrin and metalloproteinase with thrombospondin motifs (ADAMTS) in the cell.^51^, ^52^ The destruction of type II collagen (Col2A1) promotes the hypertrophy of chondrocytes through the bone morphogenetic protein (BMP) pathway, thus exacerbating the progression of TMJ osteoarthritis.^53^ In addition, mineralization of cartilage has been found to be involved in the development of TMJ osteoarthritis.^10^ The signalling molecules involved in the regulation of ECM degeneration are summarized below.

The role of β‐catenin, which regulates MMP13 and ADAMT5, is controversial in TMJ osteoarthritis. Compressive mechanical force reduces endogenous β‐catenin and leads to ECM degradation. These pathological changes can be recovered by restoring the β‐catenin signalling.^54^ However, other authors found that mice with conditional β‐catenin activation showed TMJ osteoarthritis‐like phenotypes and up‐regulation of MMP13 and ADAMT5 expression.^21^, ^55^

The Notch cascade consists of Notch and Notch ligands, which are involved in regulating the proliferation, differentiation, maturation and apoptosis of chondrocytes.^56^ Regulation of this cascade (Notch1/Jagged1/Hes5) promotes the expression of MMPs and reduces the expression of tissue inhibitor of metalloproteinase‐1 (TIMP‐1) in the cell.^28^, ^32^, ^57^

The activation of α2A‐adrenergic receptor signals via the extracellular regulated protein kinases 1 and 2 (ERK1/2) and protein kinase A (PKA) pathways stimulates the production of matrix degradation associated enzymes, including MMP‐3 and MMP‐13.^58^ Osteopontin, an inflammatory factor, induces the expression of MMPs via the NF‐κB signalling pathway.^59^

The HTRA1‐DDR2‐MMP‐13 axis plays an important role in ECM degradation.^27^ This process starts with the overexpression of high–temperature requirement A1 (HTRA1) and the degradation of the pericellular matrix components, such as type VI collagen. The disappearance of pericellular matrix can cause the transmembrane protein DDR2 to be activated by Col2A1. Ultimately, DDR2 activates MMP13 and accelerates TMJ osteoarthritis.^27^, ^60^

### 3.3 Remodelling of subchondral bone in TMJ osteoarthritis

Abnormal subchondral bone remodelling is one of the first pathological features and clinical signs of TMJ osteoarthritis.^3^ In the initial stage of TMJ osteoarthritis, the subchondral bone predominantly exhibits bone loss, while slow repair activities increase bone mass at the subchondral plate in the late stage.^61^, ^62^ The loss of subchondral bone in the early stages of TMJ osteoarthritis contributes to cartilage degeneration and the onset of osteoarthritis.^4^ Increases in the stiffness and thickness of the condylar osteochondral interface (the region that covers the calcified cartilage and subchondral cortical bone) at the late stage of TMJ osteoarthritis are caused by the calcification of cartilage and the formation of subchondral cortical bone.^62^, ^63^

It is worth noting that abnormal subchondral bone remodelling in TMJ osteoarthritis is induced by decreased osteoblast and increased osteoclast activities.^4^, ^62^ The WNT5A/receptor tyrosine kinase‐like orphan receptor 2 (Ror2) pathway was found to promote the migration and differentiation of osteoclast precursors via the Ca^2+^/nuclear factor of activated T‐cells (NFAT) pathway.^64^ In addition, overexpression of transforming growth factor‐β1 (TGF‐β1) increased the uncoupling of osteoclastic and osteoblastic activity and led to abnormal changes in subchondral bone.^19^, ^20^ The activation of α2A‐adrenergic and β2‐adrenergic receptors via neurotransmitters could destroy the subchondral bone by inducing osteoclast maturation via the RANKL pathway.^58^, ^65^

The role of sex hormones, such as oestrogen and progesterone, in TMJ osteoarthritis is still unclear.^66^, ^67^ oestrogen is involved in the formation of condylar fibrocartilage and subsequently maintains cartilage stability by inhibiting the Wnt pathway through oestrogen receptor‐α.^68^ The level of oestrogen is associated with the severity of TMJ osteoarthritis. In the early stage of TMJ osteoarthritis, a high level of oestrogen exerts protective effects by inhibiting osteoclast activity and reversing the abnormal absorption of subchondral bone.^66^ In contrast, a low level of oestrogen may increase the severity of TMJ osteoarthritis.^69^ However, the results of different studies are inconsistent. Some studies have suggested that oestrogen promotes MIA‐induced subchondral bone erosion by activating oestrogen receptor‐β.^70^ Interestingly, a high level of progesterone exerts a protective effect by reducing the degeneration of subchondral bone by inhibiting NF‐κB activity.^67^

## 4 THE PERSPECTIVES ON HYPERTROPHY AND ANGIOGENESIS IN TMJ OSTEOARTHRITIS

Endochondral osteogenesis‐like changes have been reported in both human osteoarthritis and experimental models of the osteoarthritic process.^71^ Chondrocyte hypertrophy and cartilage angiogenesis are important features of endochondral osteogenesis.^71^, ^72^ Interestingly, these two processes have also been shown to play an important role in TMJ osteoarthritis. Cytokines secreted by hypertrophic chondrocytes can direct and attract endothelial cells into cartilage.^39^ Increasing evidence has suggested that most hypertrophic chondrocytes have the potential to transform into osteoblasts and osteocytes, which are then involved in bone formation.^73^

### 4.1 Chondrocyte hypertrophy in TMJ osteoarthritis

Hypertrophic chondrocytes are characterized by cellular expression of Col X, MMP13 and alkaline phosphatase (ALP).^74^, ^75^ Van der Kraan et al reported that chondrocyte hypertrophy‐like changes play an essential role in the early and late stages of osteoarthritis.^76^ Hypertrophic chondrocytes highly express MMPs to degrade their surroundings, as observed in TMJ osteoarthritis. The process of hydroxyapatite deposition and the mineralization of cartilage are accelerated when Col X is present in the cartilage matrix.^77^ The matrix vesicles secreted by hypertrophic chondrocytes contain phosphatidylserine that aggregates calcium and phosphate to form mineralized nodules.^10^ In addition, the process of angiogenesis, which includes the migration and adhesion of endothelial cells, is further enhanced by hypertrophic chondrocytes in TMJ osteoarthritis.^78^

Chondrocyte hypertrophy is regulated by different molecular signalling pathways, such as the Indian hedgehog (Ihh) pathway.^15^ Calcium/calmodulin‐dependent protein kinase II (CaMKII), which up‐regulates Ihh expression, can be activated by increases in the intracellular calcium concentration. Activation of CaMK II not only promotes the expression of Ihh but also alleviates the inhibitory effect of parathyroid hormone receptor (PTH1R) on chondrocyte hypertrophy.^79^ Activation of PTH1R inhibits chondrocyte hypertrophy via an Ihh‐PTHrP negative feedback pathway, thereby maintaining the balance between chondrocyte proliferation and hypertrophy.^80^, ^81^ The expression of FGFR3 inhibits Ihh, but this effect is diminished in TMJ osteoarthritis.^23^ Ihh transmits information to the nucleus through the Ihh‐Smo‐Gli signalling pathway.^15^, ^77^ The pathway ultimately induces chondrocyte hypertrophy via Runt‐related transcription factor 2 (Runx2), a transcription factor that directly regulates the expression of Col X and MMP13.^76^, ^77^, ^82^, ^83^ In addition, an increased intracellular calcium concentration can promote chondrocyte hypertrophy through calcium‐sensing receptor (CaSR) in the endoplasmic reticulum.^84^

Col2A1, the main component of the ECM, can be degraded by hypertrophic chondrocytes in the context of TMJ osteoarthritis. A reduction in collagen in the ECM, in turn, induces chondrocyte hypertrophy.^53^ In a disease‐free model, Col2A1 activated integrin β1 (ITGB1). In addition to their interaction with Col2A1, ITGB1 receptors compete with BMP for SMAD1 binding and then inhibit SMAD1 activation and nuclear transport. The loss of Col2A1 promotes TMJ osteoarthritis by activating the BMP‐SMAD1 signalling pathway and increasing the expression of Runx2 and Col X.^53^

The Wnt family is another critical signalling pathway that regulates chondrocyte hypertrophy in TMJ osteoarthritis. The canonical Wnt pathway promotes Runx2 and Col X synthesis via β‐catenin. This pathway can be activated during TMJ osteoarthritis by the down‐regulation of DNA methyltransferase 3B (Dnmt3b).^21^, ^55^, ^85^ In addition to the canonical Wnt pathway, the non‐canonical Wnt pathway induces chondrocyte hypertrophy and migration via the c‐Jun N‐terminal kinase (JNK) signalling pathway.^86^

**FIGURE 3:**
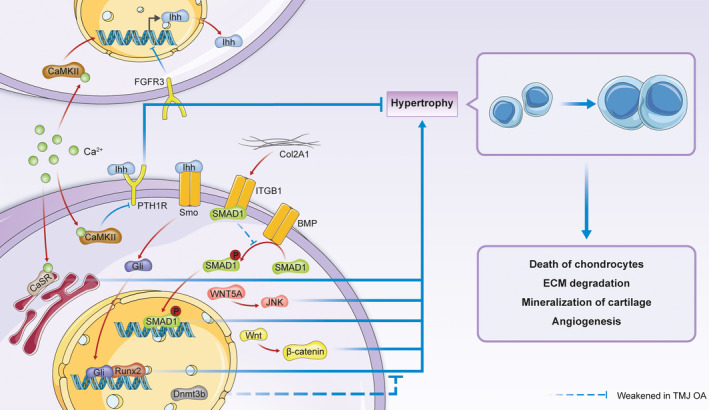
Molecular mechanism of chondrocyte hypertrophy in TMJ osteoarthritis

Figure 3 summarizes some of the related signalling pathways that are critical to chondrocyte hypertrophy.

### 4.2 Angiogenesis promotes the development of TMJ osteoarthritis

Studies have revealed that angiogenesis can promote the development of osteoarthritis.^87^, ^88^ Wang et al found that the number of newly formed blood vessels at the osteochondral junction was increased in mice with TMJ osteoarthritis‐like changes.^89^ These new blood vessels can transport inflammatory mediators and sustain inflammation in TMJ osteoarthritis.^90^ When new blood vessels invade the cartilage, they promote both chondrocyte hypertrophy and mineral deposition in the matrix.^88^ Osteophytes can then incorporate with the newly formed vessels on the surface of the joint to facilitate hard tissue formation through the process of endochondral osteogenesis.^88^ Neurogenesis follows angiogenesis. An increase in the formation of blood vessels and nerves between the subchondral bone and articular cartilage may mediate the pathological process of osteoarthritis and contribute to the pain associated with osteoarthritis.^12^

Vascular endothelial growth factor (VEGF) is crucial for angiogenesis and is an important mediator of TMJ osteoarthritis. Injection of VEGF led to TMJ osteoarthritis‐like changes in mice by stimulating endothelial cell proliferation and migration and stabilizing newly formed blood vessels.^91^ The expression of VEGF is up‐regulated by several transcription factors, such as hypoxia‐inducible factor‐1 (HIF‐1).^11^, ^92^ High levels of the dickkopf‐related protein‐1 (DKK‐1) protein were found in the synovial fluid of TMJ osteoarthritis patients. It has been suggested that DKK‐1 and high‐mobility group box 1 (HMGB1) direct the nuclear localization of HIF‐1 and thus promote the synthesis of VEGF.^92^, ^93^ The inflammatory factors IL‐6 and IL‐1β increase the level of VEGF by inducing VEGF transcription in the nucleus. IL‐6 activates oestrogen‐related receptor γ (ERRγ) via extracellular signal‐regulated kinase (ERK1/2). In contrast, IL‐1β directly activates NF‐κB.^94^, ^95^

Vascular endothelial growth factor stimulates cartilage neovascularization through several different downstream pathways. The Notch signalling pathway is another important pathway associated with angiogenesis, and VEGF is a positive regulatory factor of Notch. A study showed that the HIF‐1‐VEGF‐Notch1 signalling pathway mediated angiogenesis in TMJ osteoarthritis.^11^ Delta‐like ligand 4 (Dll4) also plays a key role and contributes to vascular development. VEGF can up‐regulate the expression of Dll4 by activating p‐ERK1/2 signalling, thus facilitating angiogenesis in TMJ osteoarthritis.^96^ In addition to VEGF, angiopoietins (Angs), such as Ang‐1 and Ang‐2, are overexpressed in the injured TMJ. Studies have shown that overexpression of Ang‐1 is caused by IL‐1β‐mediated activation of the MAPK pathway.^94^, ^97^

Neurovascular interactions are involved in the progression of TMJ osteoarthritis. Proangiogenic factors such as VEGF and molecules secreted by endothelial cells were found to stimulate nerve growth.^98^ The growth of nerve endings also plays a role in angiogenesis. Nerve growth factor (NGF), which regulates nerve growth, survival and repair, can stimulate angiogenesis in TMJ osteoarthritis.^99^

**FIGURE 4:**
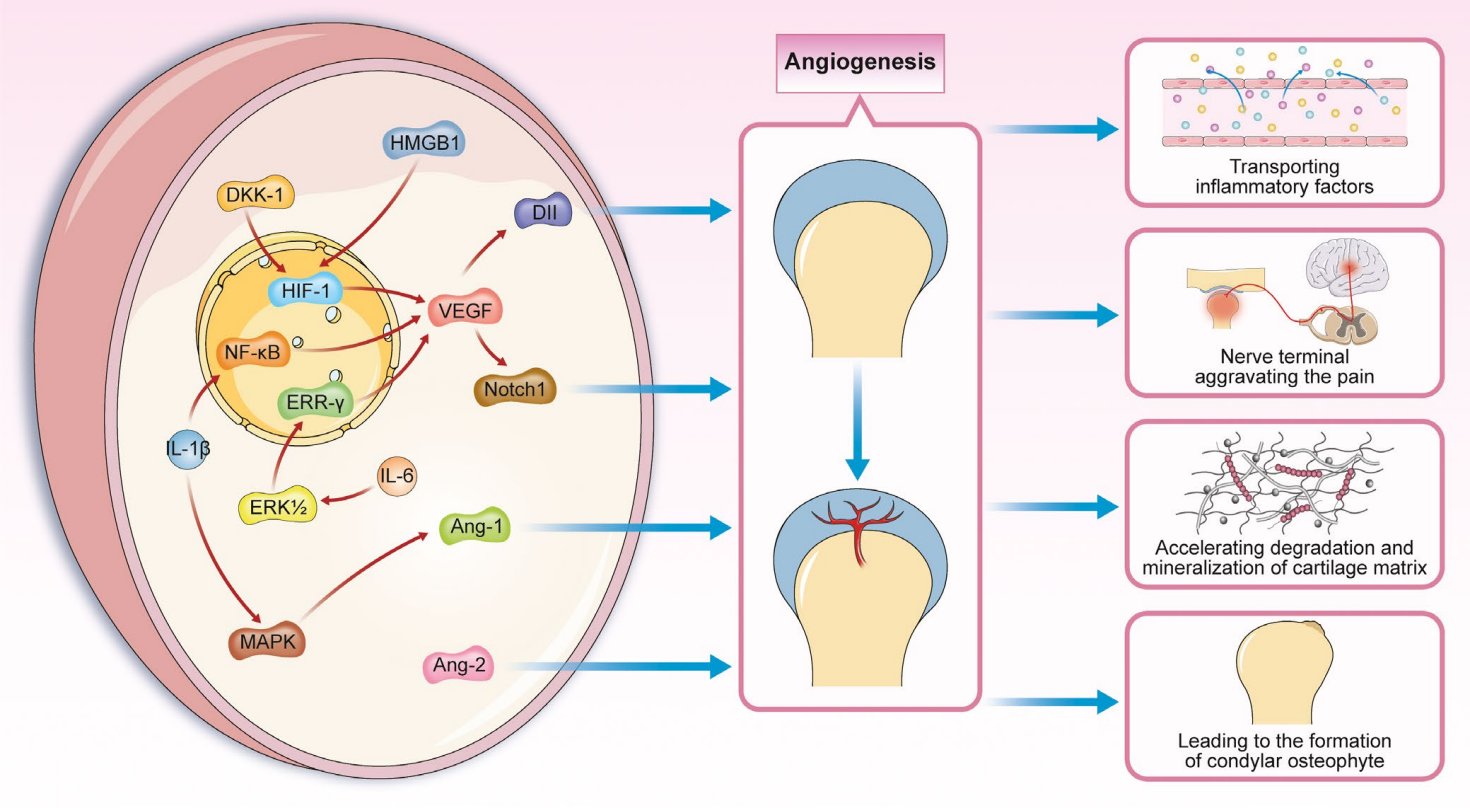
Molecular mechanism of angiogenesis in TMJ osteoarthritis

The regulatory mechanism and role of angiogenesis in TMJ osteoarthritis are shown in Figure 4.

## 5 CONCLUSION

The TMJ in animals has been successfully constructed in a variety of ways to stimulate the structural and organizational changes in osteoarthritis. Chondrocyte death, ECM degradation and subchondral bone remodelling play essential roles in TMJ osteoarthritis. Moreover, the endochondral osteogenesis‐like changes such as chondrocyte hypertrophy and endochondral angiogenesis suggest that the pathological changes of TMJ osteoarthritis are closely related to the process of osteogenesis. The molecular pathways that regulate TMJ osteoarthritis help us to better understand the pathological mechanisms of TMJ osteoarthritis. More importantly, these signalling molecules may serve as potential therapeutic targets to help find effective disease‐modifying strategies for TMJ osteoarthritis.

## AUTHOR CONTRIBUTIONS

**Baochao Li:** Conceptualization (lead); Visualization (lead); Writing‐original draft (lead). **Guangzhao Guan:** Formal analysis (equal); Project administration (equal); Writing‐original draft (supporting). **Li Mei:** Data curation (equal); Investigation (equal); Writing‐original draft (supporting). **Kai Jiao:** Project administration (equal); Validation (supporting); Writing‐review & editing (equal). **Huang Li:** Conceptualization (lead); Funding acquisition (lead); Project administration (lead); Resources (lead); Supervision (lead); Writing‐review & editing (lead).

## Data Availability

This article does not contain available data.
